# Acute Ankle Sprain Management: An Umbrella Review of Systematic Reviews

**DOI:** 10.3389/fmed.2022.868474

**Published:** 2022-07-07

**Authors:** Diego Gaddi, Angelo Mosca, Massimiliano Piatti, Daniele Munegato, Marcello Catalano, Giorgia Di Lorenzo, Marco Turati, Nicolò Zanchi, Daniele Piscitelli, Kevin Chui, Giovanni Zatti, Marco Bigoni

**Affiliations:** ^1^Orthopedic Department, San Gerardo Hospital, University of Milano-Bicocca, Monza, Italy; ^2^Department of Medicine and Surgery, University of Milano-Bicocca, Monza, Italy; ^3^Department of Paediatric Orthopedic Surgery, University Hospital Grenoble-Alpes, University Grenoble-Alpes, Grenoble, France; ^4^Transalpine Center of Pediatric Sports Medicine and Surgery, University of Milano-Bicocca, Monza, Italy; ^5^Hospital Couple Enfant, Grenoble, France; ^6^School of Physical and Occupational Therapy, McGill University, Montréal, QC, Canada; ^7^Department of Kinesiology, University of Connecticut, Storrs, CT, United States; ^8^Department of Physical Therapy, Waldron College of Health and Human Services, Radford University, Roanoke, VA, United States

**Keywords:** rehabilitation, treatment, management, acute, ankle, sprain, injury

## Abstract

Even though ankle sprains are among the most frequent musculoskeletal injuries seen in emergency departments, management of these injuries continues to lack standardization. Our objective was to carry out an umbrella review of systematic reviews to collect the most effective evidence-based treatments and to point out the state-of-the-art management for this injury. PubMed, Scopus, Web of Science, and the Cochrane library were searched from January 2000 to September 2020. After removing duplicates and applying the eligibility criteria, based on titles and abstracts, 32 studies were screened. At the end of the process, 24 articles were included in this umbrella review with a mean score of 7.7/11 on the AMSTAR quality assessment tool. We found evidence supporting the effectiveness of non-surgical treatment in managing acute ankle sprain; moreover, functional treatment seems to be preferable to immobilization. We also found evidence supporting the use of paracetamol or opioids as effective alternatives to non-steroidal anti-inflammatory drugs to reduce pain. Furthermore, we found evidence supporting the effectiveness of manipulative and supervised exercise therapy to prevent re-injury and restore ankle dorsiflexion.

## Introduction

During sports activities, especially indoor sports (1) (e.g., volleyball, basketball, and dance), acute ankle sprains are very common, with considerable diagnostical and treatment costs for healthcare systems and high socioeconomic impact due to absenteeism from work (2). Epidemiological data have shown that ~80% of individuals will suffer an ankle sprain during their lifetime (3, 4). This injury is more frequent in women than in men; likewise, children are more affected than adolescents and adults: the progressive development of coordination patterns and neuromuscular control plays a protective role (5–8).

Less than 15% of acute ankle sprains are coupled with a fracture of the ankle or foot, suggesting that the damage mainly concerns soft tissues. Recently, Romero-Morales et al. (9) showed a decreased thickness of the plantar fascia in individuals with lateral ankle sprain when compared with healthy subjects. X-rays are required in 77–99% of cases (10, 11). Based on the anatomical classification, we can identify three types of ankle sprain patterns, namely, lateral, syndesmotic, and medial. Ankle sprains involve the lateral ligament group (~85%), because it is less resistant to load, so it is easier to injure compared with the others ligament groups (12). The lateral ankle ligament is composed of the anterior talofibular ligament (ATFL), the calcaneofibular ligament (CFL), and the posterior talofibular ligament (PTFL) (10). The ATFL is the weakest and when it is damaged it causes an antero-posterior instability, while the involvement of the CFL turns into an inversion instability. In emergency departments, a clinical classification is more valuable. We can divide acute ankle sprain into three different grades (Table 1): grade I injuries ligaments are only stretched without macroscopic tearing; grade II injuries show partial tear of ligaments, frequently with a complete tear of the ATFL and an additional partial tear of the CFL; and finally grade III injuries are associated with complete tear of ligaments with disruption of both the ATFL and CFL, capsular tear could be associated.

**Table 1:** Classification of lateral ankle sprain based on increasing ligamentous damage and morbidity.

**Grade**	**Hematoma/swelling/pain**	**Anterior drawer test**	**Talar tilt test**	**Anatomic lesion**	**Stability**
I	Positive	Negative	Negative	Incomplete tear of ATFL	Stable
II	Positive	Positive	Negative	Complete tear of ATFL, incomplete tear of CFL	Unstable
III	Positive	Positive	Positive	Complete tear of ATFL, complete tear of CFL	Unstable

*ATFL, anterior talofibular ligament; CFL, calcaneofibular ligament (12)*.

Although several studies on acute ankle sprain are available, there is a considerable variety of classifications, follow-up times, treatments, outcome measures, and endpoints. Ankle sprain treatment ranges from physical therapy [e.g., orthoses, functional exercise, strength, and endurance (13)] to pharmacology support and orthopedic surgery. Thus, it is difficult to propose a standard algorithm to manage and treat an acute ankle sprain when it occurs in the emergency department. Moreover, a systematic review of published clinical practice guidelines for the treatment of acute lateral ankle sprains revealed that their quality was poor (14). Therefore, this study reviews literature on the different treatments proposed for acute ankle sprain. Specifically, by means of an umbrella review of systematic reviews, we analyze and describe the state-of-the-art management of this injury.

## Materials and Methods

### Focused Question Based

This study is an umbrella review of systematic reviews. Following the Preferred Reporting Items for Systematic Review and Meta-analysis (PRISMA) (15) guidelines, we developed a specific foreground question that focused on the management of ankle sprains. The following PICO was formulated: Population: adults with acute ankle sprain; Intervention and Comparison: conservative and surgical treatments; Outcomes: pain, swelling, range of motion, instability, function, reinjury rate, and return to sport (see Section Search Strategy and Study Selection for additional details).

### Eligibility Criteria

We only considered articles that satisfy the following eligibility criteria: (i) the study must be a systematic review, with or without a meta-analysis; (ii) it must assess the suitability of an intervention for the treatment or prevention of ankle sprains; and (iii) the population examined must not include chronic ankle instability. We have excluded studies dealing exclusively with epidemiology, etiopathogenesis, and diagnosis of ankle sprains. We have also excluded studies concerning pediatric patients. We have included all types of treatments.

### Search Strategy and Study Selection

We conducted a comprehensive literature search on PubMed, Scopus, Web of Science, and the Cochrane library from 2000 up to September 2020. We included only systematic reviews and meta-analyses in order to analyze the highest level of evidence and to increase the external validity of the present study.

**Figure 1:**
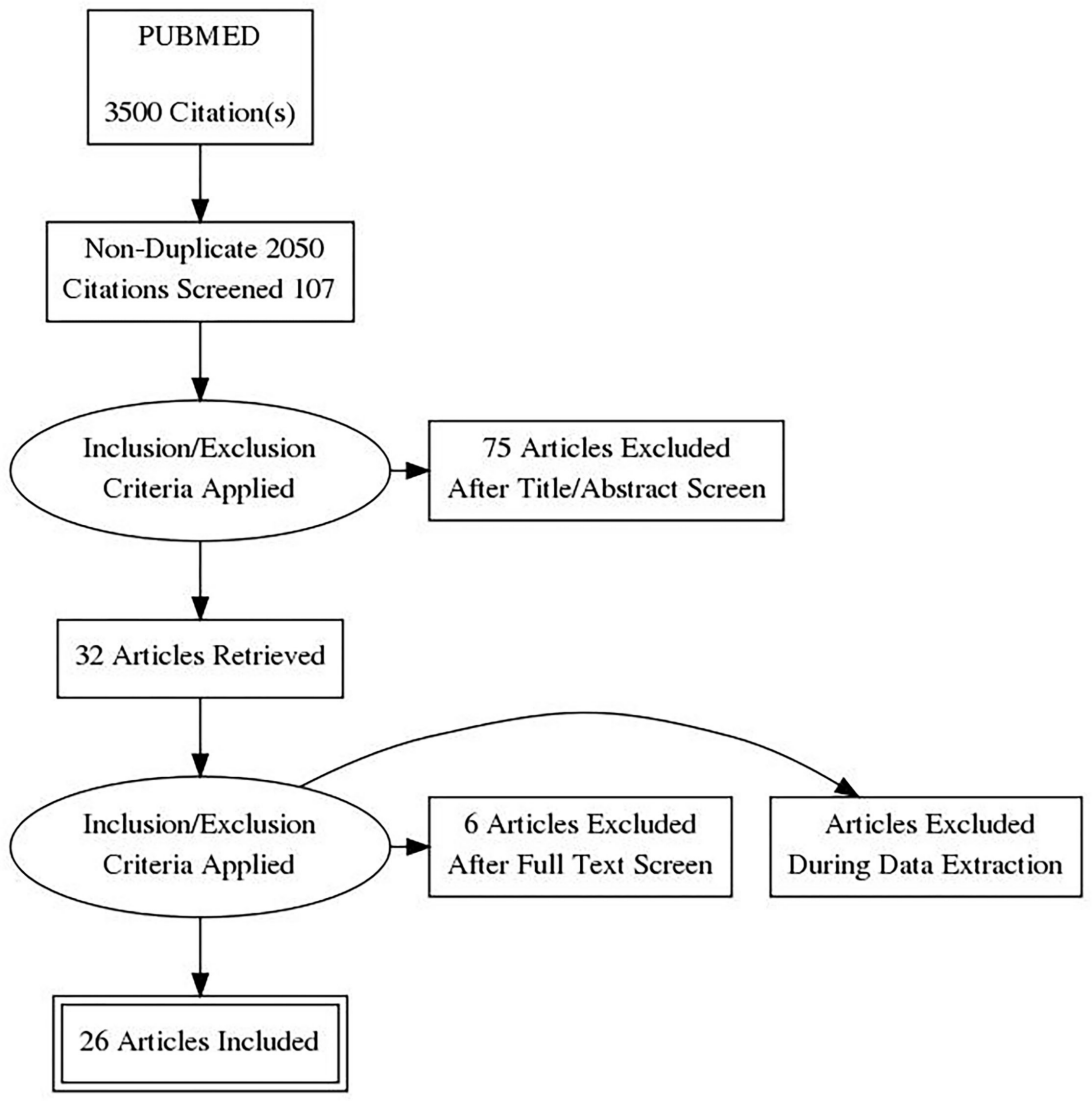
PRISMA flow diagram.

We developed our research using the following keywords: (a) “ankle” AND “sprain”; (b) “ankle sprain” AND “management”; (c) “ankle sprain” AND “treatment”. No fields in the search engines were specified in order to expand the research (e.g., in PubMed the word “treatment” was translated by the search engine as “therapeutics” [MH] OR “therapeutics” [All Fields] OR “treatments” [All Fields] OR “therapy” [Subheading] OR “therapy” [All Fields] OR “treatment” [All Fields] OR “treatment's” [All Fields]). As a result, we found 3,500 citations. Different authors screened all the articles found, first removing duplicates, and then applying the eligibility criteria based on titles and abstracts; the 32 remaining studies were checked a second time by reading them and applying eligibility criteria to identify a list of relevant articles. Any disagreement was resolved by discussion among all the authors (Figure 1). Another eight studies were then removed, leaving a total of 24 articles for this umbrella review.

Regarding treatments, we considered both conservative and surgical strategies. Among non-surgical interventions, we reported acute treatment [e.g., RICE (rest, ice, compression, elevation) therapy, immobilization, taping, and bracing], drugs [including NSAIDs (non-steroidal anti-inflammatory drugs) and other medications], rehabilitation and manual therapy (e.g., proprioceptive, coordination and strength training, supervised exercises, and manipulative therapy), and complementary therapies (e.g., acupuncture and electrical stimulation). We selected these categories according to a literature review. Different outcomes have been assessed, including pain, swelling, range of motion, instability, function, and reinjury rate. In our study, we also focused on return to sport.

### Methodological Study Quality Assessment

Two authors (DG and MC) independently assessed the quality of the systematic reviews using the AMSTAR tool (16). Any disagreement was resolved by group discussion and consensus.

Consistent with the methods used by Doherty et al., we decided to assess the review as “high” or “low” quality, based on a score of 7/11 on the AMSTAR quality rating. This quality threshold was used to conduct the best evidence synthesis of reviews (17).

## Results

### Study Selection

We performed our research following PRISMA guidelines (15), as shown in Figure 1. In the beginning, we collected 3,500 studies. At the end of the process, only 24 systematic reviews met the eligibility criteria and were therefore included, with a mean AMSTAR score of 7.7/11 (Table 2 shows the quality of the selected articles, first author, and year of publication).

**Table 2:** Quality assessment of the included studies according to the AMSTAR scale.

	**AMSTAR criteria**											
**References**	**1**	**2**	**3**	**4**	**5**	**6**	**7**	**8**	**9**	**10**	**11**	**Total**
Al Bimani et al. (38)	1	1	1	1	1	1	1	1	1	1	1	11
Bleakley et al. (26)	1	0	1	0	0	1	1	1	0	0	0	5
Bleakley et al. (23)	1	0	1	0	0	1	1	1	1	0	0	6
Brantingham et al. (27)	1	1	1	1	1	1	1	1	0	1	1	10
Feger et al. (35)	1	1	1	0	0	1	1	1	0	0	0	6
Jones and Amendola (18)	0	0	1	1	0	1	0	0	0	0	0	3
Jones et al. (24)	1	1	1	1	1	1	1	1	1	1	1	11
Kemler et al. (19)	0	0	1	0	1	1	1	1	1	0	1	7
Kerkhoffs et al. (20)	1	1	1	1	1	1	1	1	1	0	1	10
Kerkhoffs et al. (33)	1	1	1	0	1	1	1	1	1	0	1	9
Kim et al. (36)	1	1	1	1	1	1	1	1	1	0	1	10
Loudon et al. (28)	1	0	1	0	0	1	1	1		1	0	6
Ortega-Avila et al. (21)	1	1	1	1	1	1	1	1	1	1	1	11
Park et al. (37)	1	1	1	1	0	1	1	1	1	0	1	9
Struijs and Kerkhoffs (34)	1	1	1	1	0	0	1	1	0	0	0	6
Tassignon et al. (39)	1	1	1	1	1	0	0	0	0	1	1	7
Terada et al. (29)	1	0	1	1	0	1	1	1	0	0	0	6
van den Bekerom et al. (25)	1	1	1	0	1	1	1	1	1	0	0	8
van den Bekerom et al. (22)	1	1	1	1	1	1	1	1	1	1	1	11
van der Wees et al. (30)	1	1	1	1	1	1	1	1	1	1	0	10
van Os et al. (31)	1	1	1	0	0	1	1	0	1	0	0	6
van Rijn et al. (32)	1	0	1	0	0	1	1	1	1	0	1	7
Vancolen et al. (40)	1	1	1	0	1	1	1	1	1	0	0	8
Wikstrom et al. (41)	1	1	1	0	1	0	0	0	0	0	0	4

All the studies considered investigated treatment strategies for acute ankle sprains. Six reviews investigated specifically acute treatment (18–23) (AMSTAR range 3–11). Two reviews evaluated drug therapy (24, 25) (AMSTAR range 8–11). Seven reviews evaluated rehabilitation and manual therapy (26–32) (AMSTAR range 5–10). Two reviews evaluated surgical treatment (33, 34) (AMSTAR range 6–9). Three reviews analyzed other treatments (35–37) (AMSTAR range 6–10). Finally, four reviews considered return to sport (38–41) (AMSTAR range 4–11). Table 3 summarizes the results and conclusions of included studies, grouped based on the intervention type. While several studies could have been assigned to more than one strategy, each review was noted once in Table 3, and the assignment was based on the primary focus of the review.

**Table 3:** Results of the best evidence synthesis from the reviews.

**References**	**AMSTAR**	**Outcome**	**Intervention**	**Results**	**Conclusion**
**Acute treatment**					
Bleakley et al. (23)	6	Pain, swelling, ROM	Cryotherapy	Application of ice in addition to exercise seems to be effective.	More high-quality studies are needed to reach a strong conclusion.
Jones and Amendola (18)	3	Time to return to preinjury activity, subjective instability, reinjury, subjective satisfaction	Immobilization compared with early functional treatment	Functional treatment is superior to immobilization for return to joint instability and reinjury rate preinjury activity.	Early functional treatment seems to be superior to immobilization.
Kemler et al. (19)	7	Reinjury, pain, swelling, instability, function	External support	Bracing in comparison to other forms of external support is better in terms of functional outcomes.	Future studies should be about the differences between different types of ankle brace.
Kerkhoffs et al. (20)	10	Pain, swelling, joint instability, reinjury	Immobilization	Functional treatment is superior to immobilization for multiple outcomes.	Functional treatment seems to be the best option.
Ortega-Avila et al. (21)	11	Pain, rapid recovery to functional capacity	Conservative treatment (e.g., RICE, cryotherapy, exercise, manual therapy)	After the application of conservative treatments in most cases, significant improvement in terms of pain relief and return to functional capacity was achieved	Conservative treatment decreases pain and allow a fast return to functionality
van den Bekerom et al. (22)	11	Pain, swelling, ROM	RICE	There is no evidence about the effectiveness of RICE therapy.	National guidelines and experience should guide treatment.
**Drugs**					
Jones et al. (24)	11	Pain, swelling, adverse events, self-reported function	NSAIDs compared to opioids and paracetamol	It seems that opioids and paracetamol are equivalent as painkillers to NSAIDs. Moreover, paracetamol could have less gastrointestinal side effects compared to NSAIDs.	Future studies should focus on selective COX-2 NSAIDS, comparing them to non-selective NSAIDs and paracetamol.
van den Bekerom et al. (25)	8	Pain, swelling, adverse events	Oral and topical NSAIDs compared to placebo	Both oral and topic NSAIDs are superior to placebo in treating acute ankle sprain symptoms.	Authors support NSAIDs for the initial treatment for acute ankle sprains, despite the sample size of selected studies.
**Rehabilitation and manual therapy**					
Bleakley et al. (26)	5	Pain, function, swelling, reinjury	NSAIDs, manual therapy, neuromuscular training, electrophysical agent, complementary	NSAIDs reduce pain and improve ankle function. Neuromuscular training decreases functional instability and minimizes re-injury. Manual therapy techniques improve ankle range of motion (mobility)	The combination of manual therapy, NSAIDs and neuromuscular training improves ankle function and prevent re-injury.
Brantingham et al. (27)	10	Subjective function, ROM, pain, swelling, proprioception, stabilometry	Manual therapy	It seems that manipulative therapy combined with exercises could help treat ankle sprain.	More trials are needed to clarify the effectiveness of manual therapy
Loudon et al. (28)	6	ROM, pain, swelling, stabilometry, gait parameters	RICE compared with RICE and manual therapy	Ankle range of motion and pain are improved by manual therapy both in case of acute and chronic ankle sprains.	Manual therapy seems to ameliorate ankle function, more studies are needed to establish the clinical relevance of these results.
Terada et al. (29)	6	Dorsiflexion	Manual therapy compared with therapeutic modalities or exercises or psychological interventions	To restore ankle range of mobility, it is important to include static-stretching intervention.	Ankle dorsiflexion improvement has to be considered important clinical outcomes during the ankle sprain care pathway.
van der Wees et al. (30)	10	Reinjury, postural stability, ROM	Exercise therapy and manual mobilization	Exercise therapy was effective in reducing the risk of recurrent sprains after acute ankle sprain. No effects of exercise therapy were found on postural sway in patients with functional instability. Four studies demonstrated an initial positive effect of different modes of manual mobilization on dorsiflexion ROM.	It is likely that exercise, including the use of a wobble board, is effective in the prevention of recurrent ankle sprains. The manual mobilization has an initial effect on dorsiflexion ROM, but the clinical relevance of these findings for physiotherapy practice may be limited.
van Os et al. (31)	6	Return to sport, pain, swelling, instability, ROM	Conventional treatment compared to supervised rehabilitation	Adding supervised exercises to conventional treatment is not support by strong evidence.	More trials are needed to define the role of supervised exercise clearly.
van Rijn et al. (32)	7	Pain, instability, reinjury	Supervised exercises compared to conventional treatment	It seems that the addition of supervised exercises to conventional treatment leads to faster and better recovery and a faster return to sport	Additional supervised exercises are recommended together with conventional treatment in patients with an acute ankle sprain.
**Surgery**					
Kerkhoffs et al. (33)	9	Reinjury, pain, instability	Surgical vs. conservative strategies	Evidence is not sufficient to define the relative effectiveness of surgical and conservative strategies	Conservative strategies seem to be the best choice, given the risk of operative complications and the higher costs associated to surgery.
Struijs and Kerkhoffs (34)	6	Symptoms improvement, reinjury rate, instability, activity level	Immobilization vs. functional treatment vs. surgery vs. ultrasound vs. diathermy vs. ice vs. homeopathy vs. physical therapy	Immobilization, functional treatment and surgery are superior to placebo in improving outcomes	Immobilization is superior to functional treatment and surgery in improving symptoms, whereas functional treatment and surgery are superior in ameliorating stability and return to activity
**Other treatments**					
Feger et al. (35)	6	Pain, function, edema	Electrical stimulation	Electrical stimulation does not raise the outcomes studied.	Evidence is insufficient to support the use of electrical stimulation.
Kim et al. (36)	10	Self-reported function, reinjury	Acupuncture	No evidence is found about the effectiveness or safety of acupuncture treatments, both alone and in combination with other treatments.	Future studies are needed to test the safety and effectiveness of acupuncture.
Park et al. (37)	9	Pain	Acupuncture	Acupuncture seems to appear useful in order to decrease acute ankle sprain symptoms. No adverse events were found.	Evidence is insufficient to recommend acupuncture.
**Return to sport**					
Al Bimani et al. (38)	11	Return to sport	Functional treatment, mobilization, NSAIDs	Functional treatment, compressing stockings, joint mobilization, hyaluronic acid injection, jump stretch flex band program and NSAIDs seem to short the period to return to sport.	Return to sport seem to be influenced by a variety of factors; however, results should be interpreted carefully due to the heterogeneity of articles collected
Tassignon et al. (39)	7	Return to sport	Functional treatment	No studies propose a clear paradigm to return to sport after lateral ankle sprain injury. So, the authors propose a list of factors, that could be useful to build a hypothetic algorithm.	There are no published algorithms that guide return to sport after lateral ankle sprain injury. Several factors that could influence RTS, are presented.
Vancolen et al. (40)	8	Return to sport	Operative treatment vs. non-operative treatment	Overall, an average of 93.8% of athletes were able to return to sport at the preinjury level.	Both operative and non-operative treatment provide a high rate of return to the preinjury level of sport after a syndesmotic ankle injury.
Wikstrom et al. (41)	4	Return to sport	Functional treatment	The consensus was found for sport-specific movement, whereas partial agreement for static balance, patient-reported outcomes, range of motion, and strength.	RTS should be guided by static balance, patient-reported outcomes, range of motion, and strength.

### Acute Treatment

Bleakley et al. (23) (AMSTAR 6) included 22 studies involving 1,469 participants to analyze the effectiveness of cryotherapy to manage acute soft-tissue damage. Concerning acute ankle sprain, there is marginal evidence that cryotherapy is effective if it is added to exercises during the management of the early stages of this injury. They do not find consensus about cryotherapy duration and the method with which ice should be used (23).

Jones and Amendola (18) (AMSTAR 3) included 9 studies involving 920 subjects to compare immobilization to early functional treatment. They reported that functional treatment allows earlier return to sport and to work, while there is no substantial difference concerning instability and re-injury rate, although studies seem to favor early functional treatment.

Kemler et al. (19) (AMSTAR 7) included nine studies involving 1,250 patients to compare braces to other functional treatment types. They found no differences concerning time to return to pre-injury activities, time to reduce symptoms, re-injury, and joint instability rates, but it seems that braces have better functional outcomes using the Foot and Ankle Outcome Score and Karlsson scoring scale.

Kerkhoffs et al. (20) (AMSTAR 10) included 22 studies involving 2,157 participants to analyze immobilization as a treatment for acute ankle sprains. They found that functional treatment has better results considering time to return to pre-injury activities, swelling reduction, joint stiffness, and subjective and objective joint instability. They did not find any differences for recurrence or pain.

Ortega-Avila et al. (21) (AMSTAR 11) included 20 studies, involving 2,236 subjects, to demonstrate that conservative treatment (e.g., RICE, cryotherapy, exercise, and manual therapy) effectively manages pain and functional recovery.

van den Bekerom et al. (22) (AMSTAR 11) included 11 studies, involving 868 patients to examine the evidence about RICE therapy during the management of acute ankle sprain. Even if it seems that early post-traumatic immobilization is beneficial, they do not find sufficient evidence to support the effectiveness of RICE therapy.

### Drugs

Jones et al. (24) (AMSTAR 11) included 20 studies involving 3,305 subjects to compare the ability of paracetamol, opioids, and NSAIDs to reduce acute ankle sprain symptoms (e.g., pain, loss of function, and swelling) and side effects. The most important finding is that NSAIDs and paracetamol are equivalent in reducing pain at a 3-day follow-up; they also found no statistically significant differences in pain relief between NSAIDs and opioids. They concluded with a high grade of uncertainty that NSAIDs seem to reduce swelling and allow a faster return to normal activities than paracetamol and opioids, but more studies are needed to confirm these results. Opioids cause more gastrointestinal and neurological side effects than NSAIDs, while NSAIDs induce more gastrointestinal side effects than paracetamol.

van den Bekerom et al. (25) (AMSTAR 8) included 28 studies involving 3,447 patients to analyze the effectiveness of oral and topical NSAIDs to treat acute ankle sprains. No trials compared the effectiveness of oral and topical routes of delivery. The most important finding is that independent of the pharmaceutical form, NSAIDs effectively decrease pain and swelling at least on short-term follow-up.

### Rehabilitation and Manual Therapy

Bleakley et al. (26) (AMSTAR 5) included 23 studies, involving 3,027 patients to analyze if the combination of conservative strategies in addition to supervised exercises with external support could increase functional outcomes after acute ankle sprains. The most important finding is that independent of the pharmaceutical form, the NSAIDs effectively decrease pain and swelling on short-term follow-up. Moreover, manual therapy seems to improve ankle range of motion if applied in the early phases of an acute ankle sprain; finally, supervised neuromuscular training appears to decrease the re-injury rate.

Brantingham et al. (27) (AMSTAR 10) included 19 studies, involving 2,363 patients, to examine the state-of-the-art supporting manipulative therapy of the lower limb. According to these authors, manipulative therapy and supervised exercises are effective for short-term treatment of acute ankle sprain.

Loudon et al. (28) (AMSTAR 6) included 8 studies, involving 244 patients to determine the effectiveness of manipulative therapy in cases of lateral ankle sprains. According to these authors, manual therapy seems to improve ankle range of motion and decrease pain.

Terada et al. (29) (AMSTAR 6) included 9 studies, involving 196 patients to estimate the effectiveness of treatments to restore dorsiflexion after an acute, recurrent, and chronic ankle sprain. According to these authors, static-stretching for triceps surae included in a standardized rehabilitation program may improve ankle dorsiflexion after an acute sprain.

van der Wees et al. (30) (AMSTAR 10) included 17 studies involving 2,376 patients to analyze supervised exercises and manipulative therapy outcomes in patients with acute ankle sprain or instability. The most important finding is that exercise therapy helps to prevent recurrent ankle sprains. Moreover, manual mobilization seems to positively effect restoring ankle range of motion.

van Os et al. (31) (AMSTAR 6) included 7 studies, involving 436 patients, to compare supervised rehabilitation training to conventional treatment to manage acute lateral ankle sprains. It seems that supervised rehabilitation training added to conventional treatment may have better outcomes when compared to conventional treatment alone.

van Rijn et al. (32) (AMSTAR 7) included 11 studies involving 776 patients to determine the effectiveness of supervised rehabilitation training added to conventional treatment compared with conventional treatment alone. The authors reported that supervised rehabilitation training may produce a shorter recovery period and an earlier return to sports activities.

### Surgery

Kerkhoffs et al. (33) (AMSTAR 9) included 20 studies involving 2,562 patients to compare conservative treatment to surgical treatment in acute lateral ankle sprain injury. The evidence reviewed was insufficient to determine the relative effectiveness of surgical and conservative strategies for managing acute lateral ankle sprains.

Struijs and Kerkhoffs (34) (AMSTAR 6) included 38 studies involving 9,976 patients to analyze ankle sprain treatments. They found that surgery was able to decrease the number of patients who did not return to sports and who develop clinical instability.

### Other Treatments

Feger et al. (35) (AMSTAR 6) included 4 studies involving 162 participants, but they do not find any statistically significant results to support the effectiveness of electrical stimulation in reducing symptoms or improving functional outcomes.

Kim et al. (36) (AMSTAR 10) included 20 studies involving 2,012 subjects. When comparing acupuncture vs. no acupuncture, and acupuncture vs. another non-surgical intervention, they do not find any statistically significant results.

Park et al. (37) (AMSTAR 9) included 17 studies involving 1,820 patients to examine the effectiveness of acupuncture to reduce patients' global symptoms and pain, especially as an add-on treatment. However, they do not recommend using this practice due to the limited number of studies and insufficient high-quality evidence.

### Return to Sport

Al Bimani et al. (38) (AMSTAR 11) included 14 studies involving 1,142 patients to find a consensus about influencing factors for return to play after an acute ankle sprain is treated conservatively. According to these authors, many factors should be considered as influential factors in return to play.

Tassignon et al. (39) (AMSTAR 7) failed to find studies that establish algorithms to define the process to return to sport (RTS) for patients with a lateral ankle sprain, so they proposed variables that could be used for criteria-based RTS decision paradigm.

Vancolen et al. (40) (AMSTAR 8) included 10 studies, involving 333 patients to evaluate the rate of RTS after a syndesmotic ankle injury. According to the authors, most patients can return to sport at the preinjury level in both non-operative and operative treatment groups.

AMSTAR 4 included 11 studies to find consensus among expert opinions about items that have to be considered to develop an RTS criterion for the management of lateral ankle sprain. They found several important criteria, including sport-specific movement, static balance, patient-reported outcomes, range of motion, and strength.

## Discussion

Despite the fact that acute ankle sprains are among the most commonly seen injuries in the emergency department, a standardized protocol for its management has not been established. Furthermore, the heterogeneity of interventions and outcomes examined in this revision, and the range in quality of these studies make it difficult to propose a standard algorithm for acute ankle sprain management. Based on this umbrella review, non-surgical treatment is effective to manage acute ankle sprains, and functional treatment seems to be superior when compared to immobilization. Moreover, paracetamol and opioids are as effective as NSAIDs in reducing pain, thus they represent an alternative treatment option. Manipulative and exercise therapy could be also recommended, especially during the initial recovery phase, to prevent reinjury, and to restore dorsiflexion.

### Acute Treatment

After an acute ankle sprain, during the first few days (48–72 h), the most used treatment protocol is RICE therapy, which consists of a combination of rest, ice, compression, and elevation. Rest reduces the metabolic tissue demand, decreasing the amount of blood circulating in the damaged area. Ice induces vasoconstriction, it brings down the temperature, diminishing the metabolic rate of cells. It also fights against exudate formation and hemorrhaging, which are responsible for the swelling. Finally, ice has an analgesic role, which is also used in the rehabilitation phase in order to facilitate exercises. Compression limits the swelling and stops hemorrhaging, while elevation improves lymph drainage and venous circulation (22, 23). Although several guidelines promote this approach, there are few studies that demonstrate the effectiveness of this therapy, in part due to several difficulties that exist when researchers try to create a study protocol (22). For example, most patients apply ice before going to the hospital and there are many different ways to use cryotherapy, including mode, duration, and frequency of application (23).

After the acute medical care, various treatments exist to manage the acute ankle sprain. Nevertheless, they can be classified into three main groups, namely, (1) conservative or conventional, which includes immobilization with cast or splint, (2) functional, which consists of early mobilization added to external supports, such as tapes, elastic bandages, and braces, in order to protect the joint from the risk of re-injury, as well as coordination training, and (3) surgery (20).

According to our revision, functional treatment represents a better choice than conservative treatment: fewer patients suffer from swelling, ROM limitations, joint stiffness, and joint instability. Moreover, patients are more satisfied, and they return earlier to their pre-injury work and sports activities; thus, the associated social costs could also be reduced (18–21). As far as recommending a functional treatment as the first choice to manage an acute ankle sprain, there are few studies that compare different types of external supports, even if it seems that some types of braces could be superior based on patient outcomes (19). We, therefore, recommend that more studies are needed to understand which types of external support to use with functional treatment.

### Drugs

To reduce swelling, reduce pain, and decrease the time to return to work, NSAIDs are the most commonly used medications in the treatment of ankle sprains, as they have both analgesic and anti-inflammatory effects. Swelling and pain are mediated by the inflammation process, which is also a part of healing; NSAIDs, however, can impair this process. The most common side effect is gastrointestinal bleeding, but they can cause other problems such as bronchospasm or renal failure. The advent of COX-2 selective NSAIDs has recently decreased gastrointestinal side effects at the cost of higher cardiovascular risk (24). NSAIDs are usually prescribed in an oral or topical form, which are both more effective than a placebo. However, we have not found studies comparing the two different formulations (25). Other drugs, customarily used, are paracetamol and opioids, alone or combined with each other, which does not have an anti-inflammatory effect. Opioids act centrally and peripherally on their specific receptors, while the mechanism of paracetamol is not fully clear, although it seems to involve several central pathways, including prostaglandin, serotonergic, nitric oxide, and cannabinoid ones. Paracetamol is hepatotoxic, while opioids are known to cause nausea, respiratory depression, sedation, vomiting, constipation, and dysphoria. Regarding pain, there is no evidence of a difference between paracetamol and NSAIDs; however, paracetamol seems to have fewer gastrointestinal side effects. Considering pain, NSAIDs and opioids would be equivalent, but NSAIDs would have fewer gastrointestinal and neurological side effects. NSAIDs seem to reduce swelling and help regain function better than paracetamol and opioids, but according to our review, we need more studies to confirm these results with a higher grade of certainty (24). We recommend the use of paracetamol or opioids as effective alternatives to NSAIDs for pain reduction. More studies are needed that focus on the impact of COX-2 selective NSAIDs and different pharmaceutical formulations of these drugs.

### Rehabilitation and Manual Therapy

Several ankle sprain rehabilitation programs are available with different methods and exercises, practiced alone or in combination with other specific treatments. Functional treatment is the combination of an external device that supports the ankle with a rehabilitation program, which includes early joint mobilization. Many functional treatment programs exist, but it is not clear which program produces the best outcomes including preventing reinjury, and whether supervised exercises are superior to conventional treatment. Conventional treatment includes all types of external support, which allow at most partial joint mobility, combined with standard non-supervised exercises, application of ice, and partial weight-bearing (30). Instead, this conventional treatment could be combined with rehabilitation supervised by a physical therapist that usually begins within 2 weeks from the injury, and emphasizes balance training and coordination exercises. According to our review, no strong evidence was found demonstrating the superiority of supervised exercises to conventional treatment, especially if we consider outcome measures at short-term follow-up (27, 32).

Restoring the ankle dorsiflexion of the ankle and preventing joint stiffness are key goals of rehabilitation programs because the loss of range of motion is a risk factor for recurrent sprains and chronic joint instability. Different interventions and rehabilitation programs are proposed, including proprioceptive training, coordination training, strength training, functional exercises, and manual mobilization. However, the most effective method of ensuring such a restoration is yet to be determined (30).

According to our review, the effectiveness of manual therapy to improve ankle dorsiflexion seems to be limited to the early stages of rehabilitation (30). Its effects have to be linked to increasing triceps surae flexibility with static-stretching exercises, which could also be performed at home, decreasing the need for treatment in terms of time (29).

However, at the same time, it is important to keep in mind that many factors contribute to limitations in ankle range of motion, such as pain, muscle spasm, and swelling, so analgesics, myorelaxants, and anti-oedema drugs, as well as manipulative therapy and lymphatic drainage may also help to restore ankle dorsiflexion. Therefore, a multimodal approach is needed to establish which factors are reducing the range of motion in order to select the most appropriate interventions (28, 29).

Motor control plays a key role in walking and feed forward postural mechanisms (8, 42, 43); however, following an ankle sprain, lower limb muscle activation patterns may be altered (44), thus another popular intervention is neuromuscular training, particularly using the wobble balance board in the sub-acute phase, to improve proprioception that seems to prevent re-injury (26).

### Surgery

Surgical treatment of acute ankle sprain generally consists of primary repair of torn ligaments with suturing. Kerkhoffs et al. (33) analyzed trials comparing surgical interventions with conservative management and most of the evidence found was not sufficient to demonstrate effectiveness. However, they found some evidence that may demonstrate the superiority of surgery to improve stability as objectively measured by a positive talar tilt or positive anterior drawer sign on stress radiographs; nevertheless, the clinical relevance of these differences remains unclear. Moreover, surgery is associated with an increased risk of stiffness and limitations in ankle mobility, higher costs, and additional complications associated with surgery itself, including infection and deep vein thrombosis. It should be noted that ankle and foot surgery showed growing interest over the last decade (45, 46).

Struijs and Kerkhoffs (34) found that surgery is superior to immobilization with respect to RTS and objectively decreasing instability. At the same time, however, they did not find relevant differences in clinical and functional outcome measures. Similarly, no differences were found when surgery was compared with functional treatment.

Due to the inconclusive and conflicting data about the efficacy and safety of surgical intervention, in our opinion, more studies are needed before recommending surgery as the first option of treatment; however, we think that surgery should be considered when patients with persistent symptoms such as chronic ankle joint instability are non-responsive to other treatments.

### Other Treatments

Alternative medicine practices such as acupuncture and electrical stimulation have also been used to treat acute ankle sprains.

Electrical stimulation is an electrical current produced from an external device that is applied to the body using an electrode. The electrical current can depolarize sensory or motor nerve fibers, curb the formation of edema, and facilitate tissue healing. In theory, different types of electrical stimulation could be used, such as high-voltage pulsed electrical stimulation and neuromuscular electrical stimulation. High-voltage pulsed electrical stimulation is used to suppress edema development by modifying cell permeability. In contrast, neuromuscular electrical stimulation is used to facilitate muscle contractions, which in turn improves venous and lymphatic return and decreases edema. According to our revision, no evidence exists to support the effectiveness of electrical stimulation in the management of acute ankle sprains (35).

Concerning acupuncture, some studies would advocate its effectiveness, especially as an add-on treatment during pain management in grade I and II ankle sprains; moreover, it seems to be more effective than a placebo. Nevertheless, these results are based on subjective parameters such as patient-reported global symptom improvement or patient-reported functional assessment and studies with a few patients, so results can be overestimated. Furthermore, no study demonstrates a statistically significant result when acupuncture was compared with NSAIDs as a single treatment or add-on treatment. Moreover, almost all studies are conducted in China, where acupuncture is an ancient medical technique that is widely integrated into their culture and healthcare system, so further research is needed to establish the acceptance of this practice in other countries (36, 37).

Acupuncture may represent another pain management option to reduce the use of medications; therefore, more clinical trials are required to examine the effectiveness of acupuncture to treat acute musculoskeletal injuries, especially as an add-on treatment, and to test the acceptance of this practice in cultures outside of China. Moreover, in our opinion, it will be important to analyze the efficacy of acupuncture using objective parameters and performance-based functional scores.

### Return to Sport

Many athletes develop long-term symptoms, defined as chronic ankle instability, and it is still debated whether early RTS plays a role in this. The key concerns with chronic ankle instability are the increased risk of reinjury and its sequelae such as post-traumatic osteoarthritis, instability, stiffness, muscle hypotonicity, pain, swelling, and functional loss and disability (47, 48). The range of time to RTS is often based on the severity of the injury. A typical grade I lateral ankle sprain may take 2–6 weeks to RTS and a more severe syndesmotic injury takes an average of 46.4 days to RTS (40).

According to our review, many studies separately investigate influencing factors for RTS, but they failed to determine a paradigm (38). The decision about RTS should include balance, proprioception, strength, range of motion, agility tests, and psychological stress. It is important to evaluate both intrinsic modifiable risk factors, such as balance, endurance, muscle strength, range of motion, and extrinsic risk factors, such as the playing surface or environmental conditions (39). An opinion shared by many experts is that a crucial element when determining an athlete's readiness for RTS is the need to assess sport-specific movements, and consequently, each practitioner is encouraged to establish an objective scale to standardize lateral ankle sprain RTS decisions (41). The idea that the time since the injury (trauma) is the primary criterion for determining RTS readiness has been overcome.

In the same way, the grading of injuries based on physical exam findings (e.g., special tests) or the histological damage seen on diagnostic images is of limited value when determining RTS, especially considering the inherent difference between these injuries and the different types of treatment athletes receive. A definition that unifies these criticalities considers the RTS as a dynamic decision-making model, organized in three consecutive phases, which are based on the recovery of each individual athlete. These three phases are identified as (49).

- “return to participation,” in which the athlete is allowed to train but not perform.
- “return to sport,” in which the athlete performs but not at the levels desired before trauma.
- “return to performance,” in which an athlete returns to their pre-injury level and which is considered the goal condition.

Future studies should investigate the different variables that could influence RTS and develop a decision paradigm based on objective criteria and thresholds, possibly introducing a different paradigm based on the specific sport examined. Moreover, in adults older than 40 years who could suffer from osteoarthritis, strategies for reducing central pain mechanisms should be considered (50), taking into account that validated questionaries are available to clinicians to measure several personal factors [e.g., fear avoidance beliefs (51) and pain catastrophizing (52)].

### Limitations

Our review has some potential limitations such as articles not identified due to publication bias or bad indexing. According to the eligibility criteria, clinical practice guidelines were not included, thus we may have not included nested systematic reviews developed during the clinical guideline process. We have not considered articles written in languages, which are not English. The risk of bias of the included systematic reviews and meta-analyses has been performed using the AMSTAR tool; other instruments, e.g., the ROBIS tool (53), could be used for this purpose. We decided to administer the AMSTAR tool for its feasibility, reliability, and validity (16).

## Conclusion

There is an abundance of literature that examines ankle sprains, but results are often inconclusive and difficult to compare and contrast. In this article, we evaluated the most common treatments for acute ankle injuries and assessed the level of evidence for each systematic review.

There is high-quality evidence about the effectiveness of non-surgical treatment in managing acute ankle sprain. We also recommend functional treatment rather than immobilization.

Paracetamol or opioids are effective for reducing pain in this population and could be used as an alternative to NSAIDs. Manipulative and exercise therapy should be advised to prevent re-injury and restore dorsiflexion, especially if they are begun early during the initial phase of ankle sprain management.

## Data Availability Statement

The original contributions presented in the study are included in the article/supplementary material, further inquiries can be directed to the corresponding author.

## Author Contributions

Study conception: DG, DM, MP, MC, MB, GZ, and MT. Design of the study: DG, MC, MT, AM, DP, MP, DM, MB, GZ, and NZ. Literature search and data analysis: DG, MP, DM, MC, AM, GD, MT, DP, and NZ. Analysis of the results and drafting the manuscript: DG, MP, DM, MC, AM, GD, DP, MT, MB, GZ, NZ, and KC. Manuscript final revision: MT, AM, DP, MB, GZ, and KC. All authors of this article have read and approved the final version submitted.

## Conflict of Interest

The authors declare that the research was conducted in the absence of any commercial or financial relationships that could be construed as a potential conflict of interest.

## Publisher's Note

All claims expressed in this article are solely those of the authors and do not necessarily represent those of their affiliated organizations, or those of the publisher, the editors and the reviewers. Any product that may be evaluated in this article, or claim that may be made by its manufacturer, is not guaranteed or endorsed by the publisher.

## Abbreviations

ATFL: anterior talofibular ligament
CFL: calcaneofibular ligament
PTFL: posterior talofibular ligament
PRISMA: Preferred Reporting Items for Systematic Review and Meta-analysis
RICE: rest, ice, compression, elevation
NSAIDs: non-steroidal anti-inflammatory drugs
RTS: return to sport
ROM: range of mobility.
